# COVID-19 pandemic-related change in racial and ethnic disparities in exclusive breastmilk feeding during the delivery hospitalization: a differences-in-differences analysis

**DOI:** 10.1186/s12884-022-04570-w

**Published:** 2022-03-19

**Authors:** Kimberly B. Glazer, Luciana Vieira, Ellerie Weber, Joanne Stone, Toni Stern, Angela Bianco, Brian Wagner, Sarah Nowlin, Siobhan M. Dolan, Elizabeth A. Howell, Teresa Janevic

**Affiliations:** 1 Blavatnik Family Women’s Health Research Institute, Icahn School of Medicine at Mount Sinai, One Gustave L. Levy Place, New York, NY 10029 USA; 2 Department of Population Health Science and Policy, Icahn School of Medicine at Mount Sinai, One Gustave L. Levy Place, New York, NY 10029 USA; 3 The Raquel and Jaime Gilinski Department of Obstetrics, Gynecology and Reproductive Science, Icahn School of Medicine at Mount Sinai, One Gustave L. Levy Place, New York, NY 10029 USA; 4 Center for Nursing Research & Innovation, Department of Nursing, Mount Sinai Hospital, New York, NY USA; 5 Department of Obstetrics and Gynecology, Perelman School of Medicine, University of Pennsylvania, Philadelphia, PA USA

**Keywords:** Breastmilk, Breastfeeding, Infant, Nutrition, Disparities, COVID-19

## Abstract

**Objective:**

Exclusive breastmilk feeding during the delivery hospitalization, a Joint Commission indicator of perinatal care quality, is associated with longer-term breastfeeding success. Marked racial and ethnic disparities in breastfeeding exclusivity and duration existed prior to COVID-19. The pandemic, accompanied by uncertainty regarding intrapartum and postpartum safety practices, may have influenced disparities in infant feeding practices. Our objective was to examine whether the first wave of the COVID-19 pandemic in New York City was associated with a change in racial and ethnic disparities in exclusive breastmilk feeding during the delivery stay.

**Methods:**

We conducted a cross-sectional study of electronic medical records from 14,964 births in two New York City hospitals. We conducted a difference-in-differences (DID) analysis to compare Black-white, Latina-white, and Asian-white disparities in exclusive breastmilk feeding in a pandemic cohort (April 1-July 31, 2020, *n*=3122 deliveries) to disparities in a pre-pandemic cohort (January 1, 2019-February 28, 2020, *n*=11,842). We defined exclusive breastmilk feeding as receipt of only breastmilk during delivery hospitalization, regardless of route of administration. We ascertained severe acute respiratory syndrome coronavirus 2 (SARS-CoV-2) infection status from reverse transcription-polymerase chain reaction tests from nasopharyngeal swab at admission. For each DID model (e.g. Black-white disparity), we used covariate-adjusted log binomial regression models to estimate racial and ethnic risk differences, pandemic versus pre-pandemic cohort risk differences, and an interaction term representing the DID estimator.

**Results:**

Exclusive breastmilk feeding increased from pre-pandemic to pandemic among white (40.8% to 46.6%, *p*<0.001) and Asian (27.9% to 35.8%, *p*=0.004) women, but not Black (22.6% to 25.3%, *p*=0.275) or Latina (20.1% to 21.4%, *p*=0.515) women overall. There was an increase in the Latina-white exclusive breastmilk feeding disparity associated with the pandemic (DID estimator=6.3 fewer cases per 100 births (95% CI=-10.8, -1.9)). We found decreased breastmilk feeding specifically among SARS-CoV-2 positive Latina women (20.1% pre-pandemic vs. 9.1% pandemic *p*=0.013), and no change in Black-white or Asian-white disparities.

**Conclusions:**

We observed a pandemic-related increase in the Latina-white disparity in exclusive breastmilk feeding, urging hospital policies and programs to increase equity in breastmilk feeding and perinatal care quality during and beyond this health emergency.

**Supplementary Information:**

The online version contains supplementary material available at 10.1186/s12884-022-04570-w.

## Introduction

Breastfeeding is associated with myriad health benefits, including improved maternal-infant attachment, reductions in infectious and chronic disease risk for the dyad, decreased postpartum depression, and improved childhood cognitive development [1, 2]. The American Academy of Pediatrics and American College of Obstetricians and Gynecologists recommend exclusive breastmilk feeding (EBF) in the first six months of life [2–4]. While breastfeeding rates have improved nationally in the past decade, marked racial and ethnic disparities persist, even after controlling for other sociodemographic factors [5]. Non-Latina Black women have the lowest rates of breastfeeding initiation among any racial or ethnic group [1, 5, 6], and fare worse than non-Latina white women on indicators of breastfeeding duration and exclusivity [1]. Latinas are as likely to initiate breastfeeding as non-Latina white women but with shorter duration and lower rates of exclusivity [1, 6, 7]. Asian women report lower rates than white women of having ever breastfed, but higher rates of exclusivity at 6 months among those who initiate [5].

Women who breastfeed exclusively during their delivery hospitalization are more likely to continue breastfeeding exclusively through the first month of life [5], and EBF at discharge from delivery is used as an indicator of perinatal care quality [8]. The onset of the COVID-19 pandemic brought uncertainty in how to care for pregnant SARS-CoV-2 positive women, prevent transmission to neonates, and limit spread within the hospital [9–12]. Guidelines on hospital practices relevant to newborn feeding, such as infant rooming-in, skin-to-skin contact, length of stay, and support person/visitor policies, evolved as the health emergency unfolded. Further, Black and Latina women have been disproportionately affected by SARS-CoV-2 infection and the psychosocial and economic burdens of the pandemic [13–16]. This context may have influenced breastmilk feeding rates [17], but associations between the COVID-19 pandemic and EBF among racial and ethnic groups have not been examined.

Our objective was to evaluate whether the COVID-19 pandemic was associated with a change in racial and ethnic disparities in EBF at two hospitals in a major New York City (NYC) hospital system. At our institution, all women, including SARS-CoV-2 positive women, have been encouraged to room-in with infants since the late March 2020 onset of the first wave of the pandemic. We conducted a difference-in-differences (DID) analysis of EBF rates among singleton, term births, comparing the first wave of the COVID-19 pandemic to births in the previous year.

## Methods

### Participants

Electronic medical records (EMR) on 18,904 births from January 1, 2019 through July 31, 2020 were available for analysis. We followed criteria for the Joint Commission EBF quality metric [8] in restricting our study population to singleton, term (≥37 weeks of gestation) births not admitted to the neonatal intensive care unit (NICU) (*n*=15,779). Gestational age was recorded in the EMR based on best obstetric estimate.

We created a pandemic cohort of 3122 deliveries between April 1, 2020-July 31, 2020 and defined the pre-pandemic cohort of 11,842 deliveries between January 1, 2019-February 28, 2020 (total *n*=14,964). We omitted March 2020 to allow for a wash-out period when community spread was active in the NYC area, but before the peak of the first wave of infections. All methods were performed in accordance with the Declaration of Helsinki and the IRB of the Icahn School of Medicine at Mount Sinai approved the study. We followed STROBE guidelines for cross-sectional studies.

### Hospital Policy during COVID-19

Universal testing of women presenting to labor and delivery was instituted on March 26, 2020, with reverse transcription polymerase chain reaction (PCR) tests for the presence of SARS-CoV-2 [10, 18]. For deliveries in April 2020, one support person was allowed in the labor and delivery unit during birth and recovery; on April 29, an executive order extended the recommended time allowed for support persons to the duration of the delivery stay, and our health system changed its policy accordingly [10, 19]. Usual obstetric care continued including delayed cord clamping, skin-to skin contact, and direct breastfeeding with recommended hand and breast hygiene. Our institution established a policy mandating rooming-in of all infants who did not require NICU care in late March 2020. Women who tested positive for SARS-CoV-2 infection were isolated with their newborns and given information on safe breastfeeding practices with appropriate hygiene and co-location with social distancing as recommended by the Centers for Disease Control and Prevention (CDC) [20, 21]. Early discharge polices were implemented in late March, with mothers discharged on postpartum day one after vaginal delivery and postoperative day two after cesarean delivery unless longer stay was indicated [19].

### Measures

We used EMR data to ascertain all variables. Maternal race and ethnicity was self-reported on admission and classified according to Office of Management and Budget standards. We defined EBF as the infant not having received any formula supplementation during the delivery hospitalization (breastmilk only for all feeds, regardless of route of administration (breast, bottle, cup, dropper), as recorded by nursing staff). SARS-CoV-2 status was ascertained from PCR testing by nasopharyngeal swab.

We evaluated covariates including maternal age (<25, 25-34, ≥35), insurance type (private, Medicaid, Medicare, other, self-pay), pre-pregnancy body mass index (BMI<18.5, 18.5≤BMI<25, 25≤BMI<30, 30≤BMI<40, BMI≥40), parity (nulliparous, multiparous), gestational age (early term [37^0/7^-38^6/7^ weeks], full term [39^0/7^-40^6/7^ completed weeks], late term [41^0/7^-41^6/7^], and post-term [42^0/7^+ weeks]), and month of delivery to account for potential seasonal trends.

### Statistical Analysis

We evaluated sociodemographic and obstetric characteristics of the study population, comparing the pandemic versus pre-pandemic cohorts as well as births to SARS-CoV-2 positive versus negative women. We also compared characteristics among non-Latina Black (referred to throughout as Black), non-Latina Asian (Asian), non-Latina white (white), and Latina births. Comparisons used chi-square tests with a two-sided *p*-value of <0.05 for statistical significance.

We estimated a DID equation using log binomial regression. We specified main effects for the Black versus white risk difference, pandemic versus pre-pandemic cohort risk difference, and an interaction term representing the DID estimator. The DID estimator provides the additional disparity resulting from the pandemic, beyond pre-existing disparities. We repeated the model for Latina versus white and for Asian versus white births. To parse the potential direct influence on breastmilk feeding of SARS-CoV-2 infection from that of delivering during the pandemic era, we carried out the same DID analyses restricting the pandemic cohort to SARS-CoV-2 positive or negative status. Multivariable models adjusted DID estimates for maternal age, insurance type, pre-pregnancy BMI, parity, and month of delivery. The DID approach is generally robust to confounding if the balance of covariates between treatment and control groups is constant over time [22]. We excluded observations with missing values from multivariable analyses (<4% for BMI, <3% for PCR, <0.1% for age).

We evaluated the robustness of our results through multiple sensitivity analyses. First, we specified an alternate control group using a pre-pandemic time frame (April 1, 2019-July 31, 2019) with months identical to the pandemic period. Second, we used a spurious treatment group of August through December 2019. Third, we conducted an interrupted time series analysis to account for secular trends in EBF by examining whether the slope of EBF changed in the pandemic compared to the pre-pandemic period [23]. Finally, we considered the influence of the pandemic shortened length of stay policy by replicating our analyses using an EBF measure limited to feeds within 24 hours after birth.

## Results

We did not find notable changes in study characteristics over time (Table 1), nor within racial/ethnic groups, except for an older age distribution among Asian women in the pandemic versus pre-pandemic cohorts (*p*=0.023, Table 2).

**Table 1:** Characteristics of 14,964 singleton births by COVID-19 pandemic cohort and maternal SARS-CoV-2 status

	Pre-pandemic cohort 1/1/19-2/28/20 *n*=11,842	Pandemic cohort 4/1/20-7/31/20 *n*=3122^**a**^	Pandemic cohort with PCR test 4/1/20-7/31/20 *n*=3051	
			SARS-CoV-2 positive *n*=156^**b**^	SARS-CoV-2 negative *n*=2881^**b**^
**Maternal age**				
<25	1277 (10.8)	364 (11.7)	27 (17.3)	319 (11.1)
25-34	6280 (53.0)	1619 (51.9)	78 (50.0)	1507 (52.3)
35+	4284 (36.2)	1139 (36.5)	51 (32.7)	1055 (36.6)
**Race/ethnicity**				
Non-Latina Black	1306 (11.0)	374 (12.0)	25 (16.0)	342 (11.9)
Latina	1935 (16.4)	540 (17.3)	44 (28.2)	482 (16.7)
Non-Latina Asian	1245 (10.5)	349 (11.2)	4 (2.6)	339 (11.8)
Non-Latina white	6185 (52.3)	1670 (53.5)	76 (48.7)	1542 (53.5)
Other/unknown	1160 (9.8)	189 (6.1)	7 (4.5)	176 (6.1)
**Insurance**				
Private	9008 (76.1)	2389 (76.5)	102 (65.4)	2224 (77.2)
Medicaid	1892 (16.0)	523 (16.8)	42 (26.9)	465 (16.1)
Medicare	163 (1.4)	24 (0.8)	1 (0.6)	23 (0.8)
Other^c^	542 (4.6)	135 (4.3)	7 (4.5)	125 (4.3)
Self-pay	237 (2.0)	51 (1.6)	4 (2.6)	44 (1.5)
**Parity**				
Nulliparous	5872 (49.6)	1510 (48.4)	55 (35.3)	1440 (50.0)
Multiparous	5970 (50.4)	1612 (51.6)	101 (64.7)	1441 (50.0)
**Body Mass Index**				
Underweight (BMI<18.5)	259 (2.2)	59 (1.9)	2 (1.3)	55 (1.9)
Normal weight (18.5≤BMI<25)	4726 (39.9)	1202 (38.5)	45 (28.9)	1131 (39.3)
Overweight (25≤BMI<30)	3623 (30.6)	1005 (32.2)	57 (36.5)	920 (31.9)
Class 1 or 2 obesity (30≤BMI<40)	2443 (20.6)	635 (20.3)	42 (26.9)	565 (19.6)
Class 3 obesity (BMI≥40)	330 (2.8)	85 (2.7)	2 (1.3)	83 (2.9)
**Gestational age at delivery**				
Early term (37-38 weeks)	3110 (26.3)	792 (25.4)	48 (30.8)	726 (25.2)
Full term (39-40 weeks)	7686 (64.9)	2061 (66.0)	92 (59.0)	1910 (66.3)
Late term (41 weeks)	1020 (8.6)	263 (8.4)	16 (10.3)	239 (8.3)
Post-term (42+ weeks)	26 (0.2)	6 (0.2)	0 (0.0)	6 (0.2)

^a^Column percentages do not sum to 100 in all cases due to missing data; ^b^Sum of positive and negative cases is not equal to total number of births in cohort because 85 deliveries were missing SARS-CoV-2 PCR test data; ^c^Other race/ethnicity includes American Indian or Alaska Native, Native Hawaiian or Pacific Islander, and other or unspecified race/ethnicity

**Table 2:** Sample characteristics by race and ethnicity and pre-pandemic (1/1/19-2/28/20) versus pandemic (4/1/20-7/31/20) cohort

	Non-Latina Black *n*=1680			Latina *n*=2475			Asian *n*=1594			Non-Latina white *n*=7855		
	Pre-pandemic *n*=1306	Pandemic *n*=374	*p*-value	Pre-pandemic *n*=1935	Pandemic *n*=540	*p*-value	Pre-pandemic *n*=1245	Pandemic *n*=349	*p*-value	Pre-pandemic *n*=6185	Pandemic *n*=1670	*p*-value
**Age**			0.531			0.602			**0.023**			0.821
<25	242 (18.5)	77 (20.6)		352 (1.2)	103 (19.1)		26 (2.1)	5 (1.4)		534 (8.6)	150 (9.0)	
25-34	707 (54.1)	191 (51.1)		1085 (56.1)	290 (53.7)		691 (55.5)	167 (48.0)		3187 (51.5)	867 (51.9)	
35+	357 (27.3)	106 (28.3)		496 (25.7)	147 (27.2)		528 (42.4)	176 (50.6)		2463 (39.8)	653 (39.1)	
**Insurance**			0.523			0.156			0.327			0.051
Private	696 (53.3)	218 (58.3)		951 (49.2)	260 (48.2)		1062 (85.3)	310 (88.8)		5440 (88.0)	1484 (88.9)	
Medicaid	483 (37.0)	126 (33.7)		802 (41.5)	246 (45.6)		84 (6.8)	18 (5.2)		314 (5.1)	81 (4.9)	
Medicare	26 (2.0)	7 (1.9)		19 (1.0)	4 (0.7)		17 (1.4)	1 (0.3)		85 (1.4)	8 (0.5)	
Other	48 (3.7)	11 (2.9)		85 (4.4)	17 (3.2)		68 (5.5)	17 (4.9)		275 (4.5)	79 (4.7)	
Self-pay	53 (4.1)	12 (3.2)		78 (4.0)	13 (2.4)		14 (1.1)	3 (0.9)		71 (1.2)	18 (1.1)	
**Parity**			0.668			0.254			0.385			0.566
Nulliparous	624 (47.8)	174 (46.5)		892 (46.1)	234 (43.3)		802 (64.4)	216 (61.9)		2934 (47.4)	779 (46.7)	
Multiparous	682 (52.2)	200 (53.5)		1043 (53.9)	306 (56.7)		443 (35.6)	133 (38.1)		3251 (52.6)	891 (53.4)	
**BMI**			0.343			0.943			0.980			0.226
Underweight	9 (0.7)	6 (1.6)		21 (1.1)	6 (1.1)		69 (5.5)	20 (5.7)		135 (2.2)	25 (1.5)	
Normal weight	305 (23.4)	81 (21.7)		479 (24.8)	133 (24.6)		720 (57.8)	201 (57.6)		2773 (44.8)	736 (44.1)	
Overweight	405 (31.0)	120 (32.1)		579 (29.9)	174 (32.2)		310 (24.9)	86 (24.6)		1964 (31.8)	557 (7.1)	
Class 1-2 obesity	420 (32.2)	120 (32.1)		659 (34.1)	174 (32.2)		97 (7.8)	27 (7.7)		1037 (16.8)	264 (15.8)	
Class 3 obesity	113 (8.7)	26 (7.0)		101 (5.2)	27 (5.0)		7 (0.6)	1 (0.3)		74 (1.2)	25 (1.5)	
**Gestational age**			0.576			0.346			0.745			0.629
37-38 weeks	432 (33.1)	116 (31.0)		580 (30.0)	156 (28.9)		355 (28.5)	108 (31.0)		1408 (22.8)	355 (21.3)	
39-40 weeks	770 (59.0)	231 (61.8)		1208 (62.5)	353 (65.4)		811 (65.1)	222 (63.6)		4141 (67.0)	1138 (68.1)	
41+ weeks	104 (8.0)	27 (7.2)		146 (7.6)	31 (5.7)		79 (6.4)	19 (5.4)		636 (10.3)	177 (10.6)	

*BMI *body mass index, Underweight BMI<18.5, Normal weight (18.5≤BMI<25), Overweight (25≤BMI<30), Class 1 or 2 obesity (30≤BMI<40), Class 3 obesity (BMI≥40). *P*-value for Pearson chi-square test

The percentage of women who exclusively breastmilk fed increased from 33.5% in the pre-pandemic cohort to 37.7% in the pandemic cohort (*p*<0.001, data not shown in table). Rates increased among white (40.8% to 46.6%, *p*<0.001) and Asian (27.9% to 35.8%, *p*=0.004) women, and not among Black (22.6% to 25.3%, *p*=0.275) or Latina (20.1% to 21.4%, *p*=0.515) women (Table 3). The unadjusted DID estimator comparing Black and white women was 3.2 fewer cases of exclusive breastmilk feeding per 100 births (95% confidence interval [CI]=-8.8. to 2.5), indicating that there was no change in the Black-white disparity associated with the pandemic (Table 3). The covariate-adjusted DID estimator was similar. The unadjusted DID estimator comparing Latina and white women was 4.6 fewer cases per 100 births (95% CI=-9.3, 0.2). After adjustment for maternal age, prepregnancy BMI, insurance coverage, parity, and month of delivery, there was an increase in the Latina-white EBF disparity associated with the pandemic (DID estimator=6.3 fewer EBF cases per 100 births (95% CI=-10.8, -1.9). When we stratified analyses by SARS-CoV-2 status, DID estimators in the SARS-CoV-2 negative group were similar to the overall cohort (Table S1). EBF rates did not increase among SARS-CoV-2 positive women in any racial or ethnic group (Table S2). For Latinas, there was some evidence of a decrease in EBF among SARS-CoV-2 positive women.

**Table 3:** Difference-in-differences analysis of Black-white, Latina-white, and Asian-white disparities in exclusive breastmilk feeding at discharge, COVID-19 pandemic versus pre-pandemic period

	Pre-pandemic cohort (1/1/2019-2/28/2020)			Pandemic cohort (4/1/2020-7/31/2020)					
	Denominator	Cases (n)	Risk (%)	Denominator	Cases (n)	Risk (%)	Risk difference (%)	Lower 95% CL (%)	Upper 95% CL (%)
Black versus white births									
Non-Latina white	6171	2516	40.8	1668	778	46.6	**5.9**	**3.2**	**8.6**
Non-Latina Black	1303	294	22.6	372	94	25.3	2.7	-2.3	7.7
Difference			-18.2			-21.4	-3.2	-8.8	2.5
Adjusted Difference^a^							-3.0	-8.2	2.1
Latina versus white births									
Non-Latina white	6171	2516	40.8	1668	778	46.6	**5.9**	**3.2**	**8.6**
Latina	1927	388	20.1	537	115	21.4	1.3	-2.6	5.2
Difference			-20.6			-25.2	-4.6	-9.3	0.2
Adjusted Difference^a^							**-6.3**	**-10.8**	**-1.9**
Asian versus white births									
Non-Latina white	6171	2516	40.8	1668	778	46.6	**5.9**	**3.2**	**8.6**
Non-Latina Asian	1244	347	27.9	349	125	35.8	**7.9**	**2.3**	**13.5**
Difference			-12.9			-10.8	2.1	-4.2	8.3
Adjusted Difference^a^							0.5	-5.8	6.7

^a^Adjusted for maternal age (<25, 25-34, 35+), parity (nulliparous, multiparous), prepregnancy body mass index (BMI<25, BMI≥25), and insurance coverage (Medicaid, Private, Other/self-pay); Observations with missing covariate values dropped from adjusted analyses (<4% for BMI, <1% all others)

**Fig. 1:**
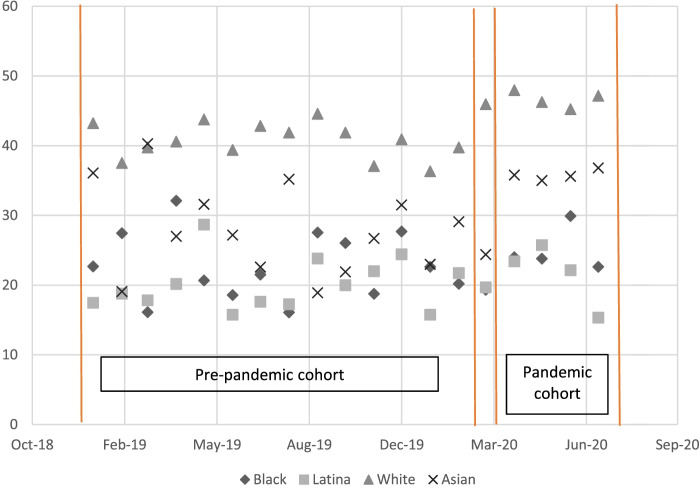
Monthly percentages of infants exclusively breastfed during the delivery hospitalization, by racial and ethnic group

Results for the increase in the Latina-white EBF disparity were robust to an alternative control (April-July 2019) cohort (DID=-5.7, 95% CI=-11.2, -0.2; Table S3). As desired, no association was found using a spurious pandemic cohort (DID=-0.2, 95% CI=-4.2, 3.7; Table S4). We did not find evidence of secular trends when examining monthly EBF percentages for each racial and ethnic group over the course of the study period (Fig. 1). The results of our interrupted time series analysis confirmed that the coefficients testing for secular EBF trends and for trend changes from the pre-pandemic to pandemic periods were not significant, so we present the DID results. Using a 24-hour EBF measure, we found a smaller but still statistically significant increase in EBF from pre-pandemic to pandemic births among White women (55.9% to 59.1%, risk difference=3.3 (95% CI 0.6-5.9) per 100 births), and the increase among Asian women was eliminated. Our finding of no change in EBF for Black and Latina women remained (data not shown).

## Discussion

The COVID-19 pandemic was associated with an increase in EBF among white and Asian women and not among Black and Latina women. Before the pandemic onset, Latina women were half as likely as white women to breastmilk feed exclusively during the delivery hospitalization; the first wave of SARS-CoV-2 infection was associated with an increase in this disparity independent of maternal sociodemographic and obstetric characteristics. Increases in EBF were limited to SARS-CoV-2 negative white and Asian women, and we found evidence of a decrease in EBF among SARS-CoV-2 positive Latina women.

Aside from preterm birth [24–26], information on COVID-19 and perinatal outcomes by race and ethnicity is scarce. EBF during the delivery hospitalization is an established perinatal quality metric [8] that has not been examined adequately in the context of COVID-19. During the onset of the pandemic, a lack of scientific clarity on the risks of maternal-neonatal transmission (whether vertical or horizontal) contributed to varying hospital practices. In turn, initial recommendations included separation of SARS-CoV-2 positive women and their infants and no direct breastfeeding. By May of 2020, the CDC indicated that vertical transmission was unlikely and reiterated the benefits of breastmilk as the ideal source of infant feeding [21]. Of note, our institution did not adopt mother-infant separation and, to the contrary, encouraged mothers and infants to room-in and continued to support breastfeeding and skin-to-skin contact with appropriate infection prevention measures.

At a NYC hospital with similar policies during the pandemic onset, 40.6% (*n*=41) of infants born to SARS-CoV-2 positive women were breastfed exclusively or mostly [27]. It is not clear, though, how study investigators defined exclusivity from the medical record. Other studies from the NYC area have reported in-hospital breastfeeding rates of 33.3%-57% among SARS-CoV-2 positive [28, 29] and 67.2% among SARS-CoV-2 negative women in-hospital [28], and 78% at 5-7 days postpartum among SARS-CoV-2 positive mothers [30]. Similarly, a hospital in Italy reported 75% breastmilk feeding among rooming-in infants with SARS-CoV-2 positive mothers [31]. However, these studies did not report exclusivity or stratify breastfeeding by race/ethnicity. The use of a strict measure of exclusive breastmilk feeding based on the nursing flow sheet in the EMR may explain the comparatively lower rates in ours compared to other studies.

While our objective was to determine whether the COVID-19 pandemic had a detrimental influence on in-hospital EBF among women of color, we instead found no change in EBF among Black or Latina women and an increase among white and Asian women. NYC was an early epicenter of the COVID-19 pandemic in the U.S.; hospitals quickly implemented policy changes [18] that may account for our results and warrant further investigation to inform interventions to support breastfeeding initiation and exclusivity. Our institution’s rooming-in policy during the pandemic may explain the increase among white and Asian women [17, 32]. Other policies such as visitor restrictions during the delivery stay or elements of the pandemic outside of hospital care may also have influenced breastmilk feeding for some groups. For example, more white and Asian women may have had employment amenable to remote work during the pandemic and the expectation of flexible work-from-home policies may have facilitated EBF intentions while in the hospital.

Another potential explanation for this association is the shorter length of stay policy enacted during the pandemic. We would expect early discharge to result in higher in-hospital EBF rates if women tend to introduce formula later in the delivery stay. Accounting for this policy attenuated some of the EBF increase among white women and all among Asian women while results were unchanged for Black and Latina women. This finding suggests that the amount of time spent in the hospital may have more of an influence on EBF at discharge among white and Asian women (i.e. they intend to breastfeed exclusively and later decide or need to introduce formula), while Black and Latina women may be more likely to mix both formula and breastmilk feeding from the outset. Since accounting for early discharge did not entirely explain the EBF increase among white women, we explored whether our findings reflected a decrease in cesarean delivery during the pandemic in a post-hoc analysis. However, adjusting for vaginal versus cesarean delivery did not attenuate the EBF increase comparing births to white women in the pre-pandemic and pandemic cohorts.

Our finding of a significant decrease in EBF among births to SARS-CoV-2 positive Latina women is potentially concerning. While estimates were based on a small number of cases, they suggest an influence of factors such as the disruption in breastfeeding education during the pandemic, when hospital group classes were discontinued. There may have been specific misperceptions about viral transmission that inhibited breastmilk feeding among Latina women, suggesting a need for more targeted patient consultation and education on safe breastmilk feeding and infant care practices in the hospital.

It is unclear why the unexpected benefit of hospital COVID-19 precautions on EBF was not observed among the Latina and Black women included in this study. We can hypothesize several mechanisms, including direct effects of maternal SARS-CoV-2 infection, the psychosocial corollaries of giving birth during the pandemic, and disparate experiences of delivery care. Rates of SARS-CoV-2 infection at delivery were higher among Black and Latina than among white women, and SARS-CoV-2 positive women may have refrained from breastmilk feeding out of fear of infecting the neonate. Latina women may also encounter language barriers, preventing access to up-to-date information about breastmilk feeding safety during the pandemic [33]. The decrease in EBF among SARS-CoV-2 positive Latina women provides some evidence in support of these explanations.

A growing literature has documented obstetric and neonatal risks of SARS-CoV-2 infection that may impact breastfeeding. Studies suggest increased risks of maternal complications [34–37], cesarean delivery [34], and preterm birth [36–39], and higher rates of admission to the intensive care unit [40–42] among pregnant women with laboratory-confirmed SARS-CoV-2 infection, as well an increase in stillbirth rates during the pandemic’s first wave in the UK [43]. Infection and pandemic-related stressors may also have reduced EBF among births to Black and Latina women, as these populations have borne a disproportionate burden of pandemic-related stress, anxiety, and food insecurity [13–15] as well as loss and emotional trauma [16]. Further, in a cross-sectional study of 237 births in our health system during the pandemic, Black and Latina women reported lower birth satisfaction than white women, which was associated with higher levels of postpartum anxiety, stress, and depressive symptoms and lower rates of EBF at discharge [19]. Parsing the influence of these potential explanatory mechanisms is a priority for further research. Our findings also emphasize the need for research investigating breastfeeding initiation, exclusivity, and duration within racial and ethnic groups, since the reasons for not breastfeeding exclusively during the delivery hospitalization appear to vary by race and ethnicity.

Our study has several limitations. Our EBF measure was subject to error or variation between clinicians or hospitals in recording infant feedings in the EMR. However, this variation would likely be consistent over time, so while absolute EBF percentages may be an under or over-estimate we expect that the change in disparity would be accurately measured. Second, although we probed potential mechanisms, we were not able to quantify the extent to which observed differences were due to changes in rooming-in, early discharge, fear of viral transmission, or another pathway. We did not have information on galactosemia or parenteral infusion, which are exclusion criteria for the Joint Commission measure, but replicated the measure as closely as possible through study restrictions. We did not analyze women of other or unknown race/ethnicity for interpretability of results. NYC was a racially diverse epicenter of the pandemic, and our results may not generalize to other settings or to hospitals with different COVID-era policies.

Strengths of our study include use of a strict EBF definition from nursing notes on all infant feedings, and ascertainment of SARS-CoV-2 PCR status from EMR lab data with universal screening of obstetric patients. We employed a robust DID design that allowed us to isolate changes in disparities associated with the pandemic. We conducted multiple sensitivity analyses, including an ITSA to test for the influence of secular trends and use of an alternate EBF measure to account for the early discharge policy.

## Conclusions

We provide a novel analysis of the implications of the COVID-19 pandemic on EBF during the delivery hospitalization, a perinatal metric with important short- and long-term health effects. We observed improvements in EBF associated with the pandemic among white and Asian but not Black or Latina women, who already face a substantial baseline disparity, and evidence of a decrease among SARS-CoV-2 positive Latina women. Changes resulted in a widening of the EBF gap between white and Latina women. Health care providers should advocate for hospital policies and programs to promote equity in breastmilk feeding, which may be particularly needed during the pandemic. Further, our findings emphasize the importance of reporting perinatal quality measures disaggregated by sociodemographic characteristics to monitor the influence of the pandemic on perinatal health care and disparities.

## Supplementary Information

**Additional file 1.**

## Data Availability

The data that support the findings of this study are available from The Mount Sinai Health System but restrictions apply to the availability of these data and so are not publicly available. Data are available from the authors (Kimberly Glazer, kimberly.glazer@mountsinai.org) upon reasonable request and with permission of The Mount Sinai Health System.

## Abbreviations

EBF: Exclusive breastfeeding
SARS-CoV-2: Severe acute respiratory syndrome coronavirus 2
PCR: Polymerase chain reaction
NYC: New York City
DID: Difference-in-differences
