# Supplementary material for: COVID-19 pandemic-related change in racial and ethnic disparities in exclusive breastmilk feeding during the delivery hospitalization: a differences-in-differences analysis
Source: BMC Pregnancy Childbirth. 2022 Mar 19;22:225. doi: 10.1186/s12884-022-04570-w (PMC8934049; doi:10.1186/s12884-022-04570-w)
Supplement: Supplementary file 1 — Additional file 1. [file 12884_2022_4570_MOESM1_ESM.docx]

**SUPPLEMENT**

**Table S1. Difference-in-differences analysis of Black-white, Latina-white, and Asian-white disparities in exclusive breastfeeding at discharge for the COVID-19 pandemic and pre-pandemic period, SARS-CoV-2 negative women**

|  | **Pre-pandemic cohort**  **(1/1/2019-2/28/2020)** | | | **Pandemic cohort**  **(4/1/2020-7/31/2020)** | | |  |  |  |
| --- | --- | --- | --- | --- | --- | --- | --- | --- | --- |
| **Outcomes** | **Denominator** | **Cases (n)** | **Risk**  **(%)** | **Denominator** | **Cases**  **(n)** | **Risk**  **(%)** | **Risk difference (%)** | **Lower**  **95% CL**  **(%)** | **Upper**  **95% CL**  **(%)** |
| Black versus white births |  |  |  |  |  |  |  |  |  |
| Non-Latina white | 6171 | 2516 | 40.8 | 1540 | 734 | 47.7 | **6.9** | **4.1** | **9.7** |
| Non-Latina Black | 1303 | 294 | 22.6 | 340 | 88 | 25.9 | 3.3 | -1.9 | 8.5 |
| Difference |  |  | -18.2 |  |  | -21.8 | -3.6 | -9.5 | 2.3 |
| Adjusted Difference^b^ |  |  |  |  |  |  | -3.7 | -9.1 | 1.7 |
| Latina versus white births |  |  |  |  |  |  |  |  |  |
| Non-Latina white | 6171 | 2516 | 40.8 | 1540 | 734 | 47.7 | **6.9** | **4.1** | **9.7** |
| Latina | 1927 | 388 | 20.1 | 479 | 109 | 22.8 | 2.7 | -1.5 | 6.8 |
| Difference |  |  | -20.6 |  |  | -24.9 | -4.3 | -9.3 | 0.7 |
| Adjusted Difference^a^ |  |  |  |  |  |  | **-5.8** | **-10.5** | **-1.1** |
| Asian versus white births |  |  |  |  |  |  |  |  |  |
| Non-Latina white | 6171 | 2516 | 40.8 | 1540 | 734 | 47.7 | **6.9** | **4.1** | **9.7** |
| Non-Latina Asian | 1244 | 347 | 27.9 | 339 | 122 | 36.0 | **8.1** | **2.4** | **13.8** |
| Difference |  |  | -12.9 |  |  | -11.7 | 1.2 | -5.1 | 7.5 |
| Adjusted Difference^a^ |  |  |  |  |  |  | -0.6 | -6.9 | 5.7 |

^a^Adjusted for maternal age, parity, prepregnancy  body mass index (BMI<25, BMI≥25), insurance coverage, and month of delivery. NTSV cesarean measure already restricted to nulliparous births so not adjusted for parity; Observations with missing covariate values dropped from adjusted analyses (<4% for BMI, <1% all others).

**Table S2. Difference-in-differences analysis of Black-white, Latina-white, and Asian-white disparities in exclusive breastfeeding at discharge for the COVID-19 pandemic and pre-pandemic period, SARS-CoV-2 positive women**

|  | **Pre-pandemic cohort**  **(1/1/2019-2/28/2020)** | | | **Pandemic cohort**  **(4/1/2020-7/31/2020)** | | |  |  |  |
| --- | --- | --- | --- | --- | --- | --- | --- | --- | --- |
| **Outcomes** | **Denominator** | **Cases (n)** | **Risk**  **(%)** | **Denominator** | **Cases**  **(n)** | **Risk**  **(%)** | **Risk difference (%)** | **Lower**  **95% CL**  **(%)** | **Upper**  **95% CL**  **(%)** |
| Black versus white births |  |  |  |  |  |  |  |  |  |
| Non-Latina white | 6171 | 2516 | 40.8 | 76 | 27 | 35.5 | -5.3 | -16.1 | 5.6 |
| Non-Latina Black | 1303 | 294 | 22.6 | 25 | 4 | 16.0 | -6.6 | -21.1 | 8.0 |
| Difference |  |  | -18.2 |  |  | -19.5 | -1.3 | -19.5 | 16.8 |
| Adjusted Difference^b^ |  |  |  |  |  |  | -9.1 | -27.4 | 9.2 |
| Latina versus white births |  |  |  |  |  |  |  |  |  |
| Non-Latina white | 6171 | 2516 | 40.8 | 76 | 27 | 35.5 | -5.3 | -16.1 | 5.6 |
| Latina | 1927 | 388 | 20.1 | 44 | 4 | 9.1 | **-11.0** | **-19.7** | **-2.4** |
| Difference |  |  | -20.6 |  |  | -26.4 | -5.8 | -19.7 | 8.1 |
| Adjusted Difference^a^ |  |  |  |  |  |  | -3.6 | -16.2 | 9.0 |
| Asian versus white births |  |  |  |  |  |  |  |  |  |
| Non-Latina white | 6171 | 2516 | 40.8 | 76 | 27 | 35.5 | -5.3 | -16.1 | 5.6 |
| Non-Latina Asian | 1244 | 347 | 27.9 | 4 | 2 | 50.0 | 22.1 | -27.0 | 71.2 |
| Difference |  |  | -12.9 |  |  | 14.5 | 27.4 | -22.9 | 77.6 |
| Adjusted Difference^a^ |  |  |  |  |  |  | -- | -- | -- |

^a^Adjusted for insurance coverage. Models did not converge with additional covariates; ^b^Adjusted for maternal age, parity, prepregnancy body mass index (BMI<25, BMI≥25), insurance coverage, and month of delivery. NTSV cesarean measure already restricted to nulliparous births so not adjusted for parity; Observations with missing covariate values dropped from adjusted analyses (<4% for BMI, <1% all others). Adjusted model not calculated for Asian-white disparity given small cell size.

**Table S3.  Results of robustness check using alternate control period (04/01/2019-07/31/2019) for the difference-in-differences analysis of the Latina-white disparity in exclusive breastfeeding at discharge**

|  | **Pre-pandemic cohort**  **(4/1/2019-07/31/2019)** | | | **Pandemic cohort**  **4/1/2020-7/31/2020)** | | |  |  |  |
| --- | --- | --- | --- | --- | --- | --- | --- | --- | --- |
|  | **Denominator** | **Cases (n)** | **Risk**  **(%)** | **Denominator** | **Cases**  **(n)** | **Risk**  **(%)** | **Risk difference (%)** | **Lower**  **95% CL**  **(%)** | **Upper**  **95% CL**  **(%)** |
| Non-Latina white | 1841 | 769 | 41.8 | 1668 | 778 | 46.6 | **4.9** | **1.6** | **8.2** |
| Latina | 541 | 111 | 20.5 | 537 | 115 | 21.4 | 0.9 | -4.0 | 5.8 |
| Difference |  |  | -21.3 |  |  | 25.2 | -4.0 | -9.8 | 1.9 |
| Adjusted Difference^b^ |  |  |  |  |  |  | **-5.7** | **-11.2** | **-0.2** |

Adjusted for insurance coverage. Models did not converge with additional covariates; ^b^Adjusted for maternal age, parity, prepregnancy body mass index (BMI<25, BMI≥25), and insurance coverage; Observations with missing covariate values dropped from adjusted analyses (<4% for BMI, <1% all others).

**Table S4. Results of robustness check using spurious treatment group (8/1/2019-12/31/2019) for the difference-in-differences analysis of the Latina-white disparity in exclusive breastfeeding at discharge**

|  | **Pre-pandemic cohort**  **(4/1/2019-7/31/2019)** | | | **False treatment cohort**  **(8/1/2019-12/31/2019)** | | |  |  |  |
| --- | --- | --- | --- | --- | --- | --- | --- | --- | --- |
|  | **Denominator** | **Cases (n)** | **Risk**  **(%)** | **Denominator** | **Cases**  **(n)** | **Risk**  **(%)** | **Risk difference (%)** | **Lower**  **95% CL**  **(%)** | **Upper**  **95% CL**  **(%)** |
| Non-Latina white | 1841 | 769 | 41.8 | 2247 | 926 | 41.2 | -0.6 | -3.6 | 2.5 |
| Latina | 541 | 111 | 20.5 | 749 | 160 | 21.4 | 0.8 | -3.7 | 5.3 |
| Difference |  |  | -21.2 |  |  | -19.9 | 1.4 | -4.0 | 6.8 |
| Adjusted Difference^b^ |  |  |  |  |  |  | -0.2 | -4.2 | 3.7 |

^a^Adjusted for maternal age (continuous), parity (nulliparous, multiparous), pre-pregnancy body mass index (BMI<25, BMI≥25), and insurance coverage (Medicaid, Private, Other/self-pay); Observations with missing covariate values dropped from adjusted analyses (<4% for BMI, <1% all other covariates)
